# The discounted value of human lives lost due to COVID-19 in France

**DOI:** 10.12688/f1000research.26975.1

**Published:** 2020-10-15

**Authors:** Joses Muthuri Kirigia, Rose Nabi Deborah Karimi Muthuri, Lenity Honesty Kainyu Nkanata, Newton Gitonga Muthuri

**Affiliations:** 1 Department of Research, African Sustainable Development Research Consortium (ASDRC), Nairobi, Kenya; 2 Faculty of Health Sciences, University of Pretoria, Pretoria, South Africa; 3 Chandaria School of Business, United States International University – Africa, Nairobi, Kenya

**Keywords:** Coronavirus disease, COVID-19, France, Gross Domestic Product, Value of human life

## Abstract

**Background: **This study estimates the total discounted value of human lives lost (TDVHL) due to COVID-19 in France as of 14 September 2020.

**Methods: **The human capital approach (HCA) model was used to estimate the TDVHL of the 30,916 human lives lost due to COVID-19 in France; i.e., assuming a discount rate of 3% and the national average life expectancy at birth of 83.13 years. To test the robustness of the estimated TDVHL, the model was rerun (a) using 5% and 10% discount rates, while holding the French average life expectancy constant; and (b) consecutively substituting national life expectancy with the world average life expectancy of 73.2 years and the world highest life expectancy of 88.17 years.

**Results: **The human lives lost had a TDVHL of Int$10,492,290,194, and an average value of Int$339,381 per human life lost. Rerun of the HCA model with 5% and 10% discount rates decreased TDVHL by Int$1,304,764,602 (12.4%) and Int$3,506,938,312 (33%), respectively. Re-calculation of the model with the world average life expectancy decreased the TDVHL by Int$7,750,187,267 (73.87%). Contrastingly, re-estimation of the model with the world’s highest life expectancy augmented TDVHL by Int$3,744,263,463 (35.7%).

**Conclusions:** The average discounted economic value per human life lost due to COVID-19 of Int$339,381 is 8-fold the France gross domestic product per person. Such evidence constitutes an additional argument for health policy makers when making a case for increased investment to optimise France’s International Health Regulation capacities and coverage of essential health services, and safely managed water and sanitation services.

## Introduction

France is one of the seven major advanced economies (G7 countries). The country has an estimated population of 64.994 million; a total gross domestic product (GDP) of Int$3,161.335 billion; and GDP per capita of Int$41,637.729 in 2020 ^1^. In 2018, approximately 10,918,992 (16.8%) of the population lived below France’s poverty threshold of €1,008 per month of disposable income ^2^. France has an inequality-adjusted human development index of 0.808 and a Gini coefficient of 32.7 ^3^.

By 14 September 2020, there were 29,182,605 coronavirus disease-19 (COVID-19) cases in the world, including 928,281 deaths, 21,027,161 recovered cases, and 7,227,163 active cases ^4^. Europe had a total of 4,080,753 COVID-19 cases, including 212,545 deaths, 2,245,583 recovered cases, and 1,622,625 active cases. On the same date, France had conducted a total of 10 million COVID-19 tests that revealed a total of 381,094 COVID-19 cases, which included 30,916 deaths, 89,059 recovered cases, and 261,119 active cases ^4^. France bore 9.3% of total cases and 14.55% of total COVID-19 deaths in Europe. France’s densities of 5,836 COVID-19 cases and 473 deaths per million population were higher than Germany’s densities of 3,117 cases and 112 deaths per million population.

Four factors might explain the relatively large number of COVID-19 deaths sustained by France. First, there was more than two months’ delay in country-wide implementation of public health interventions that could have prevented (or slowed) transmission and spread of severe acute respiratory syndrome coronavirus 2 (SARS-CoV-2). There is evidence that COVID-19 was already spreading in France by late December 2019 ^5^. However, the government only banned in mid-March 2020 gatherings of more than 100 people; the opening of non-essential public establishments; anchoring in inland and territorial waters of ships carrying more than 100 passengers; opening public establishments; opening schools and institutes of higher education; all religious gatherings; and embalming of dead bodies ^6^.

Second, the average of 13 International Health Regulations (IHR) core capacity score for France was 82 (on a scale of 0 to the target of 100) in 2019 ^7, 8^, denoting an overall IHR capacity gap of 18. As shown in Table 1, the country had IHR capacity gaps of 33 in legislation and financing, 20 in zoonotic events and the human-animal interface, 20 in food safety, 27 in laboratory, 20 in human resources, 27 in national health emergency framework, 7 in health service provision, 20 in risk communication, and 60 in points of entry ^9^. The latter gap denotes suboptimal capacity at ports/airports/ground crossings for coordination and communication of pandemic surveillance; and appropriate medical diagnosis, isolation and care of ill travellers. The French points of entry capacity score of 40 were lower than the average score for the World Health Organization (WHO) European Region (EUR) of 61 by 52.2%.

**Table 1.** International Health Regulations (IHR) capacity scores in France in 2019.

IHR capacities	France IHR capacity score	WHO European Region IHR capacity score
Legislation and Financing	67	80
IHR Coordination and National IHR Focal Point Functions	100	87
Laboratory	73	73
Surveillance	100	84
Human Resources	80	71
National Health Emergency Framework	73	73
Health Service Provision	93	73
Risk Communication	80	66
Points of Entry	40	61
Zoonotic Events and the Human-animal Interface	80	80
Food Safety	80	77
Chemical Events	100	69
Radiation Emergencies	100	77
**MEAN**	**82**	**75**

Source: World Health Organization ^9^.

Second, as shown in Table 2, generally the health system indicators for France are better than the EUR averages.

**Table 2.** Comparison of the health system and social determinants of health indicators in France with the World Health Organization European Region (EUR) averages.

Health indicators	Value in France	Average value in EUR
**Health workforce indicators (2017) ^10, 11^**		
Medical doctors per 10,000 population	32.67	34.1
Nursing and midwifery personnel per 10,000 population	114.7	81.3
Dentists per 10,000 population	6.67	5.7
Pharmacists per 10,000 population	10.64	6.8
**Medical devices indicators ^10, 11^**		
Linear accelerators per million population (2013)	7.4	N/A
Telecobalt units per million population (2013)	0.11	N/A
Radiotherapy units per million population (2013)	7.51	3.9
Magnetic Resonance Imaging per million population (2013)	10.13	N/A
**Infrastructure indicators ^10, 11^**		
Hospital beds per 10,000 population in 2018	59.1	N/A
**Essential health service coverage indicators in 2017 ^12^**		
Universal health coverage index of service coverage (UHC SCI)	**78**	**77**
UHC SCI components: Reproductive, maternal, newborn and child health	96	86
UHC SCI components: Infectious diseases	71	73
UHC SCI components: Non-communicable diseases	56	61
UHC SCI components: Service capacity and access	96	94
**Catastrophic out-of-pocket health spending (SDG indicator 3.8.2) ^10, 11^**		
Population with household expenditures on health greater than 10% of total household expenditure or income (SDG 3.8.2) in 2010 (%)	1.42	6.27
Population with household expenditures on health greater than 25% of total household expenditure or income (SDG indicator 3.8.2) in 2010 (%)	0.22	1.15
Current Health Expenditure (CHE) per Capita in Int$	5,011.2	2923
Domestic General Government Health Expenditure as % of CHE	77.09	65.0
Domestic Private Health Expenditure as % of CHE	22.91	35.0
Out-of-Pocket Expenditure as % of CHE	9.38	30.4
CHE as % Gross Domestic Product (GDP)	11.31	7.78
Domestic general government health expenditure as percentage of GDP (%)	8.72	4.92
**Social Determinants of Health**		
Population using safely managed drinking water services (%) ^10, 11^	97.85	92
Population using safely managed sanitation services (%) in 2017 ^10, 11^	88.37	68
Total labour force unemployed (%) ^1^	8.431	7.6

Source: WHO ^1, 10– 12^. Note: N/A - means not available.

The density of 32.67 medical doctors per 10,000 population was lower than the average of 34.1 for EUR by 4.38%. Nursing and midwifery personnel, dentists, and pharmacist densities in France were 29.12%, 14.54%, and 36.09%, higher than EUR averages. The French density of radiotherapy units per million population of 7.51 was 48.07% higher than the EUR average. The current health expenditure (CHE) per capita of Int$5,011.2 in France was 41.67% higher than the EUR average of Int$2,923. The out-of-Pocket expenditure as a percentage of CHE of 9.38% in France was 224%% higher than the EUR average of 30.4%. The universal health coverage (UHC) service coverage index (UHC SCI) for France was 78%, signifying a gap in coverage of essential health services of 22% ^12^. The UHC SCI component of reproductive, maternal, new-born and child health; infectious diseases; non-communicable diseases; and service capacity and access had gaps of 4, 29, 44 and 4, respectively.

About 14,298,680 (0.22%) of the population’s health spending was higher than 25% of total household income, which reflects a very high risk of catastrophic and impoverishing health care expenditures. About 97.85% and 88.37% of the French population, respectively, use safely-managed drinking-water and sanitation services ^11^; signifying that 1,397,371 (2.15%) and 7,558,802 (11.63%) people do not have access.

The type of economic evidence reported in this paper could be an essential input when health policy-makers make a case for increased investment in optimizing the IHR capacities, coverage of essential health services, and coverage of safely managed water and sanitation services to more effectively prevent and manage the current COVID-19 pandemic and future public health emergencies ^13– 21^.

A few macroeconomic studies have estimated the impact of the COVID-19 pandemic on business conditions in France ^22^. However, there is a shortage of information on the value of human lives lost due to the pandemic. This study estimates the total discounted value of human lives lost (TDVHL) due to COVID-19 in France as of 14 September 2020.

## Methods

### Ethical statement

The study relied totally upon the analysis of secondary data contained in the International Monetary Fund (IMF), WHO, Worldometer, and Santé Publique France databases that are freely available to the public. Therefore, ethical clearance was not required.

### Study location

This investigation of the value of human life was conducted on the cumulative number of persons who died of COVID-19 by 14 September 2020 in France. The study was a cross-sectional study. All the 30,916 COVID-19 people reported to have died from COVID-19 as of 14 September 2020 in France were included in the study.

### Human capital approach model

This study applied the human capital approach (HCA), initially suggested by Adam Smith in 1776 ^23^, to estimate the monetary value of human life. The Organisation for Economic Co-operation and Development ^24^ defines human capital as “ *The knowledge, skills, competencies and attributes (including stock of health) embodied in individuals that facilitate* [the] *creation of personal, social and economic wellbeing*” (p.18).

Death from COVID-19 (or any other disease or injury) extinguishes the potential of a person to tap into one’s stock of human capital either for personal development and enjoyment of leisure or to enhance societal cultural and socioeconomic wellbeing. A person’s capacity for personal development, enjoyment of life (or flourishing) ^25^, loving, religious practice, and performing expected societal roles ends upon death. It is also true that death halts individuals’ spending on the consumption of goods and services, investments, government services (including payment of fees and taxes), and imports permanently. In other words, death terminates an individual’s potential contribution to the creation of national output or GDP. Following the death of a human being at any stage of life, society losses not only the statistical person’s contribution to GDP but also other intangible contributions, e.g. child’s joy to parents, love to family and friends, companionship, fellowship, comradeship, sharing of knowledge (written or tacit) and social values.

Weisbrod ^26^ suggested measuring lost human capital as a result of premature death from any cause in terms of the deceased person’s discounted future earnings net of their consumption ^26^. In line with our past research ^13– 21^, the current study uses net GDP per capita (i.e. GDP per capita of France minus current health expenditure per person) to value human lives lost due to COVID-19 in France.

The TDVHL in France (TDVHL _FRANCE_) due to COVID-19 is the sum of DVHL among persons aged 0–14 years, 15–44 years, 45–64 years, 65–74 years, and 75 years and over ^13– 21^. Formally ^13– 21^:

$$TDVHL_{FRANCE}=\sum_{i=1}^{i=5}DVHL_{i}\;(1)$$

Where: DVHL _i_ is the discounted value of human lives lost due to COVID-19 in i ^th^ age group; i=1 is age 0–14 years, i=2 is age 15–44 years, i=3 is age 45–64 years, i=4 is age 65–74 years, and i=5 is age 75 years and over; $\sum_{i=1}^{i=5}$ is the sum of the discounted values of human lives lost in age groups denoted by number 1 to 5.

The DVHL _i_ in each of the five age groups was calculated using the following formula ^13– 21^:

$$DVHL_{i}=\sum_{t=1}^{t=n}\left(\frac{1}{{\left(1+1\right)}^{t}}\right)\times (Y_{1}-Y_{2})\times (Y_{3}-Y_{4})\times (Y_{5}\times Y_{6})\;(2)$$

Where: $\sum_{t=n}^{t=1}$ is the sum from the first year of life lost (t=1) to the last years of life lost (t=n); Y _1_ is the GDP per capita of France; Y _2_ is the current health expenditure per person in France; Y _3_ is the average life expectancy at birth in France; Y _4_ is the average life expectancy at the onset of death in the i ^th^ age group; Y _5_ is the total number of COVID-19 deaths in France as of 14 September 2020; Y _6_ is the proportion of total COVID-19 deaths borne by those in the i ^th^ age group. The baseline for the analysis is 2020.

### Data and data sources

The data analysed in this paper and the sources are contained in Table 3.

**Table 3.** Data and data sources.

Variable	Data	Data sources
Per capita GDP in France (Y _1_)	Int$41,637.729	International Monetary Fund World Economic Outlook Database ^1^
Per capita current health expenditure in France (Y _2_)	Int$5011.20068359	World Health Organization Global Health Expenditure Database ^27^
Average life expectancy at birth (ALE) in 2020 (Y _3_)	France ALE = 83.13 years; world ALE = 73.2 years; Hong Kong female ALE (world highest) = 88.17 years	Worldometer Life Expectancy Database ^28^
Average age at onset of death (Y _4_)	0–14 years = 7 years; 15–44 years = 29.5 years; 45–64 years =54.5 years; 65–74 years = 69.5 years; and 75 years and over = 75 years	Authors’ estimates
Total number of human lives lost from COVID- 19 in France by 14 September 2020 (Y _5_)	30,916	Worldometer France COVID-19 Pandemic database ^4^
Proportion of COVID-19 deaths per age group in France (Y _6_)	0–14 years = 0.000148706; 15–44 years = 0.010905125; 45–64 years = 0.103747398; 65–74 years = 0.179389313; and 75 years and over = 0.705809458.	Santé Publique France COVID-19: epidemiological update of 10 September 2020 ^29^
Proportion of COVID-19 deaths per region and territory in France	Auvergne-Rhône-Alpes = 0.088576363; Bourgogne-Franche-Comté = 0.052553664; Bretagne= 0.013274118; Centre-Val de Loire = 0.02842339; Corse = 0.002911424; Grand Est = 0.182186035; Hauts-de-France = 0.096422403; Ile-de-France = 0.383222304; Normandie = 0.022156427; Nouvelle-Aquitaine = 0.021761658; Occitanie = 0.026449544; Pays de la Loire = 0.024623736; Provence-Alpes-Côte d’Azur = 0.050185048; La Réunion = 0.0006415; Martinique = 0.000888231; Mayotte = 0.001381693; Guadeloupe = 0.001480385; Guyane = 0.002862077.	Santé Publique France COVID-19: epidemiological update of 10 September 2020 ^29^
Discount rate	3%, 5%, 10%	Related published studies ^13– 21^

### Data analysis

The human capital model was analysed using Excel 2016 software (Microsoft, New York). The study reported in this paper replicates steps that were developed and applied in our recent valuation of human life studies related to COVID-19 ^13– 15, 17– 21^.

*Step 1:* Estimation of net GDP per capita (NGDPC) as the difference between per capita GDP (PCGDP) and current health expenditure per capita (CHEPC) for France. Thus, NGDPC = PCGDP – CHEPC = (Int$41637.729 – Int$5011.20068359) = Int$36,626.53.

*Step 2:* Estimation of the undiscounted years of life lost (UYLL) from COVID-19 in France between December 2019 and 14 September 2020.

(a) Calculation of average ages of onset of death (AAOD) from COVID-19 for each of the five age groups. This entailed taking simple averages for each age group, e.g. for AAOD for 0–14 age group = (0+14)/2 = 7 years.

(b) Calculation of YLL by one person who died of COVID-19 in the age group as the difference between national average life expectancy for France and the AAOD for the specific age group. For example, YLL by a person dead in the age group 0–14 years = national average life expectancy for France (83.13 years) minus AAOD for the group (7 years) = 76.13 years. Thus, the YLL for one person who died in each of the five age groups was obtained similarly (see Table 4).

**Table 4.** Undiscounted years of life lost per person due to COVID-19 in France by 14 September 2020.

Age group (years)	(A) Average life expectancy at birth in years in France	(B) Average age at onset of death	(C) Years of life lost per person in the age group [C = A – B]	(D) COVID-19 death by age group	(E) Total years of life lost per age group [E = C x D]
0–14	83.13	7	76	4.597402597	349.40
15–44	83.13	29.5	54	337.1428571	18,205.71
45–64	83.13	54.5	29	3207.454545	93,016.18
65–74	83.13	69.5	14	5546	77,644.00
75+	83.13	75	8	21820.80519	174,566.44
**TOTAL**				**30,916**	**363,781.74**

(c) Total UYLL in each age group = UYLL per deceased person in age group multiplied by the number of persons who died in an age group. For example, total UYLL in 0-14 age group = 76 years’ x 4.5974025974026 persons dead = 349.40 undiscounted YLL.

*Step 3:* Discounting of the years of life lost (DYLL).

(a) A discount rate of 3% was chosen because it has been used in our previous COVID-19 related economic studies ^13– 21^, the economic evaluation of public health problems in Africa ^30^, the World Health Report 2000 ^31^, the burden of disease ^32^, and the World Bank Disease Control Priorities study ^33^.

(b) Calculation of the discount factors applying the discount factor formula: $\left(\frac{1}{{\left(1+r\right)}^{t}}\right)$. The discount factor for first YLL = $\left(\frac{1}{{\left(1+0.03\right)}^{1}}\right)$ = 0.970873786407767; discount factor for second YLL = $\left(\frac{1}{{\left(1+0.03\right)}^{2}}\right)$ = 0.942595909133754; discount factor for third YLL = $\left(\frac{1}{{\left(1+0.03\right)}^{3}}\right)$ = 0.91514165935316; …, discount factor for the final YLL (which is 76.13 years for 0–14 years) = $\left(\frac{1}{{\left(1+0.03\right)}^{76.13}}\right)$ = 0.105772050189903.

(c) Calculation of DYLL per deceased person in age group. DYLL in year 1 = discount factor in year one x UYLL in year one = 0.970873786407767 × 1 = 0.970873786407767. DYLL in year 2 = discount factor in year two × UYLL per person in year two = 0.942595909133754 × 1 = 0.942595909133754. DYLL in year 3 = discount factor in year three × UYLL per person in year three = 0.91514165935316 × 1 = 0.91514165935316. DYLL in the last YLL (e.g. 76 ^th^ year for 0–14 years) = discount factor in 76 ^th^ year × UYLL per person in 76 ^th^ year = 0.105772050189903 × 1 = 0.105772050189903.

(d) Estimation of total DYLL per deceased person in age group is equivalent to the sum of discount factors from year one to the last year of life.

(e) Total DYLL in each age group = DYLL per deceased person in age group multiplied by number of persons who died in age group. Therefore: UYLL in 0–14 age group = 29.80759833 × 4.597402597 = 137.04 discounted YLL (see Table 5).

**Table 5.** Discounted years of life lost per age groups due to COVID-19 in France.

Age group (years)	(A) Discounted years of life lost per life lost in age group	(B) COVID-19 death by age group	(C) Total discounted years of life lost per age group [C = A x B]
0–14	29.80759833	4.597402597	137.04
15–44	26.57766047	337.1428571	8,960.47
45–64	19.18845459	3207.454545	61,546.10
65–74	11.29607314	5546	62,648.02
75+	7.01969219	21820.80519	153,175.34
**TOTAL**		**30,916**	**286,466.96**

*Step 4:* Estimation of the total number of COVID-19 deaths in age group (COVID-19D _j_) equals the total number of COVID-19 deaths in France (TCOVID-19D) multiplied by the proportion (PROP) for that age group. For example, number of COVID-19 deaths in age group 0–14 years = TCOVID-19D × PROP = 30,916 × 0.000148706 = 4.597402597. The number of COVID-19 for each age group are in Table 6.

**Table 6.** Cumulative number of COVID-19 deaths per age group in France by 14 September 2020.

Age group (years)	(A) COVID-19 deaths in France	(B) Proportion for age group	(C) Number of deaths per age group [C = A x B]
0–14	30,916	0.000148706	4.597402597
15–44	30,916	0.010905125	337.1428571
45–64	30,916	0.103747398	3,207.454545
65–74	30,916	0.179389313	5,546
75+	30,916	0.705809458	21,820.80519
**TOTAL**			**30,916**

*Step 5:* Estimation of the discounted economic value of human lives lost due to COVID-19 in each age j ^th^ group = NGDPC × DYLL _j_ × COVID-19D _j_. For instance, DVHL for age group 0-14 = Int$36,626.53 × 29.80759833 × 4.5974025974026 =Int$5,019,209.

*Step 6:* Calculation of the share of TDVHL accruing to the 13 regions and five territories of France ^29^ through multiplication of the TDVHL by proportion of COVID-19 deaths sustained by specific region and territory.

*Step 7:* A one-way sensitivity analysis was performed to evaluate the effect of changes in discount rate and the average life expectancy on the estimated TDVHL. This entailed recalculating the HCA model with (a) 5% and 10% discount rates ^13– 21^ and (b) the world average life expectancy of 73.2 years and the world highest average life expectancy of 88.17 years, i.e. the average life expectancy of Hong Kong women ^4^. The model was reanalysed while holding all other parameters constant.

## Results

The cumulative 30,916 human lives lost from COVID-19 by 14 September 2020 in France resulted in a total of 363,781.74 undiscounted years of life lost; which was equivalent to a total of 286,466.96 discounted years of life lost.

### Findings from the HCA model: assuming a national life expectancy of 83.13 years and a 3% discount rate

Table 7 depicts the distribution by age group of the TDVHL of the 30,916 human lives lost due to COVID-19 in France by 14 September 2020.

**Table 7.** Discounted value of human life losses linked to COVID-19 in France, using the national average life expectancy of 83.13 years and a discount rate of 3% (in 2020 Int$).

Age group (years)	Discounted value of human life losses at 3% discount rate (Int$)	Average discounted value per human life lost in an age group (Int$)
0–14	5,019,209	1,091,749
15–44	328,190,849	973,447
45–64	2,254,219,824	702,806
65–74	2,294,579,538	413,736
75+	5,610,280,774	257,107
**TOTAL**	**10,492,290,194**	**339,381**

The human lives lost to COVID-19 had a TDVHL of Int$10,492,290,194, and an average value of Int$339,381 per human life lost. Out of the TDVHL, 0.05% was borne by persons aged 0–14 years, 3.13% by 15–44 years, 21.48% by 45–64 years, 21.87% by 65–74 years, and 53.47% by 75 years and above. Around 46.48% of the TDVHL accrued to persons aged 15 and 74 years. The average TDVHL decreases with increase in age of the deceased, e.g. the average TDVHL for 0–14-year-olds is three-fold that of 75-year-olds and above.

**Figure 1.**
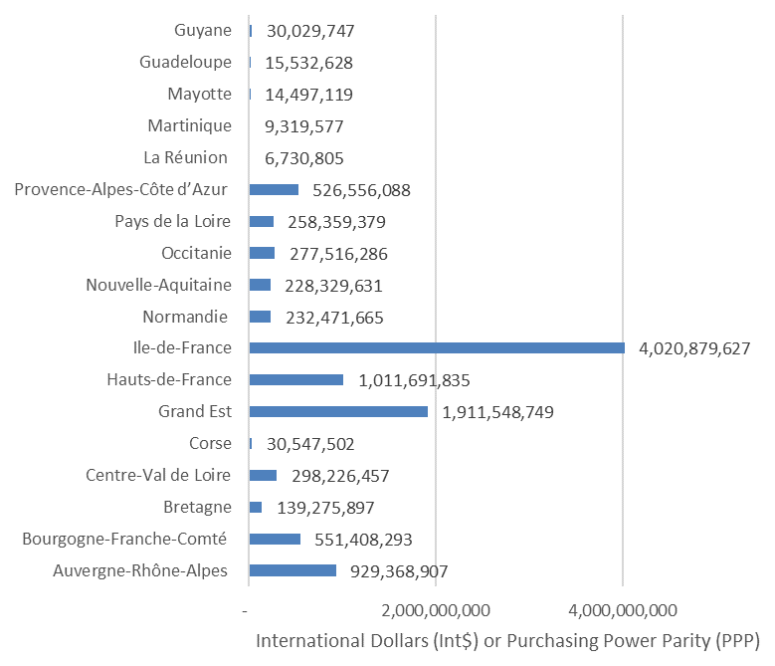
Regional and territorial distribution of the discounted value of human lives lost from COVID-19 in France as of 14 September 2020 (Int$).

***Distribution of the total discounted value of human life by regions and territories.*** Figure 1 depicts the share of TDVHL across the 13 regions and five territories of France.

About 80.3% of the TDVHL accrued to only five regions of France, i.e. Auvergne-Rhône-Alpes, Bourgogne-Franche-Comté, Grand Est, Hauts-de-France, and Ile-de-France. Grand Est, Hauts-de-France, and Ile-de-France regions alone accounted for 66.18% of the TDVHL. The territories combined made up less than 1% of TDVHL.

### Findings from the HCA model: assuming 5% and 10% discount rates holding national life expectancy and other parameters constant

Table 8 presents the effects of the application of 5% and 10% discount rates on the TDVHL due to COVID-19 in France.

**Table 8.** Discounted value of human life losses linked to COVID-19 in France, using 5% and 10% discount rates (in 2020 Int$).

Age group (years)	Discounted value of human life losses at 5% discount rate (Int$)	Discounted value of human life losses at 10% discount rate (Int$)
0–14	3,285,143	1,682,665
15–44	229,249,331	122,765,261
45–64	1,778,741,902	1,100,721,858
65–74	2,010,718,121	1,496,400,572
75+	5,165,531,095	4,263,781,526
**TOTAL**	9,187,525,593	6,985,351,882
**Discounted value** **per human life**	**297,177**	**225,946**

Rerun of the HCA model, alternately, with discount rates of 5% and 10% resulted in decreased TDVHL by Int$1,304,764,602 (12.4%) and Int$3,506,938,312 (33%), respectively. The average values per human life lost declined by Int$42,204 and Int$113,434 in turn.

### Findings from the HCA model: assuming the world and world's highest average life expectancies

Table 9 displays the impact on the TDVHL of substituting the national life expectancy with the world and world's highest average life expectancies.

**Table 9.** Discounted value of human lives lost from COVID-19 in France, assuming the world and world's highest average life expectancies (in 2020 Int$ or purchasing power parity [PPP].

Age group (years)	Discounted value of human lives lost using world average life expectancy of 73.2 years and 3% discount rate (Int$)	Discounted value of human lives lost using world highest average life expectancy of 88.17 years and 3% discount rate (Int$)
0–14	4,815,030	5,100,776
15–44	299,500,807	339,652,239
45–64	1,682,730,193	2,482,524,318
65–74	755,056,897	2,909,603,712
75+	0	8,499,672,611
**TOTAL**	2,742,102,927	14,236,553,657
**Discounted value** **per human life**	**88,695**	**460,491**

Replacement of the national life expectancy of 83.13 years with the world average life expectancy of 73.2 years in the HCA model led to decreases in the total and average TDVHL of Int$7,750,187,267 (73.87%) and Int$250,685, respectively. Contrastingly, application of the world’s highest life expectancy of 88.17 years augmented total and average TDVHL by Int$3,744,263,463 (35.7%) and Int$121,111

## Discussion

### Recap of key findings

- The 30,916 human lives lost to COVID-19 in France by 14 September 2020 had a TDVHL of Int$10,492,290,194, which is equivalent to 0.332% of France’s GDP.
- The average value was Int$339,381 per human life lost, which is 8-times the GDP per capita for France in 2020.
- Rerun of the HCA model with discount rates of 5% and 10% decreased TDEVHL by Int$1,304,764,602 (12.4 %) and Int$3,506,938,312 (33%), respectively.
- Reanalysis of the HCA model with the world average life expectancy dwindled the TDVHL by Int$7,750,187,267 (73.87%). Instead, a recalculation with the world highest average life expectancy of 88.17 years augmented TDVHL by Int$3,744,263,463 (35.7%).

### Contrasting of study findings with those from other countries

The sensitivity analysis revealed that growth in discount rate triggers contraction in the TDVHL, and an upsurge in average life expectancy amplifies the TDVHL. The two findings are consistent with those of our previous studies conducted in Brazil ^14^, Canada ^15^, China ^16^, Germany ^17^, Iran ^13^, Spain ^18^, Turkey ^19^, the United Kingdom (UK) ^20^, and the United States of America (USA) ^21^.

The China ^16^ and Spain ^18^ average values of Int$356,203 and Int$470,798 per human life loss associated with COVID-19 were 4.96% and 38.72% higher than the French average of Int$339,381. On the other hand, the French average economic value per human life of Int$339,381 was higher than those of Brazil of Int$99,629 ^14^, Canada of Int$231,217 ^15^, Germany of Int$132,960 ^17^, Iran of Int$165,187 ^13^, Turkey of Int$228,514 ^19^, the UK of Int$225,104 ^20^, and the USA of Int$292,889 ^21^ by 70.64%, 31.87%, 60.82%, 51.33%, 32.67%, 33.67%, and 13.70%, in that order. Our previous studies have attributed the differences to variations in underlying population age distributions, the YLL, the GDP per capita, and the per capita health spending ^13– 15, 17– 21^.

### Practical implications of the study findings

Evidence on the economic value of human lives losses associated with COVID-19 may be useful to the Ministry of Public Health when advocating within the Government of France for sustaining or increasing investments into the national health system, disease surveillance and response system (including IHR core capacities), and other systems (e.g. water and sanitation) that tackle social determinants of health in the pursuit of the United Nations Sustainable Development Goal 3 to “Ensure healthy lives and promote well-being for all at all ages” and Goal 6 to “Ensure availability and sustainable management of water and sanitation for all” (p.14) ^34^. Of course, the economic evidence reported in this paper is meant to complement the International Bill of Human Rights obliging the Government of France to assure every citizen’s realization of the right to life (Article 3) and to “..a standard of living adequate for the health and well-being of himself and of his family, including food, clothing, housing and medical care and necessary social services… (Article 25)” (p.76) ^35^.

### Suggestions for further economic studies

- Comprehensive studies on the multidimensional impact of COVID-19 on household’s wellbeing.
- Wide-ranging studies on the multi-sectoral impact of COVID-19 pandemic once the pandemic is eradicated.
- Consumer choice behaviour analysis in respect to uptake of various COVID-19 prevention interventions, e.g. handwashing with soap, use of safely managed drinking water and sanitation, use of face masks, and patronage of alcohol bars during COVID-19.
- Economic evaluations of cost and consequences of preventive interventions (including personal hygiene, physical distancing, safely managed human waste disposal, contact tracing, vaccines), diagnostics, and potential treatments for COVID-19. Where feasible, cost-effectiveness, cost-utility, and cost-benefit analyses ought to be designed and conducted alongside ongoing and envisaged clinical, and effectiveness randomized trials ^30, 36^.

### Limitations of the study

First, HCA has been criticized for valuing non-market contributions to society at zero dollars ^37^. For instance, traditional HCA values YLL among children below 14 years ^38^, retired (62 years and above) ^38^, homemakers (not employed outside the home), unemployed, and severely handicapped. In order to avoid discrimination against these vulnerable groups, which goes against the 1948 United Nations Universal Declaration of Human Rights ^35^ and the Constitution of the World Health Organization ^39^, YLL at all the age groups were valued at equal net GDP per capita. 

Second, the current study did not compare the costs and benefits of a raft of alternative preventive community-level interventions implemented by the French Government and citizens to limit transmission of COVID-19, e.g. bans on gatherings of more than 100 people, all religious gatherings, all travel, ships carrying more than 100 passengers, and embalming; closure of most public establishments, all schools and institutions of higher learning; and mandatory mask-wearing in public places ^40^. It was also outside the scope of the current study to appraise the costs and benefits of various options for diagnosis of COVID-19, contact tracing, quarantine, and management of persons who test positive for COVID-19 .

Finally, Stiglitz, Sen and Fitoussi ^41^ have criticized GDP for not measuring economic wellbeing (or quality of life), ignoring income inequalities, and disregarding environmental damage caused by production processes.

## Conclusion

The discounted value per human life loss of Int$339,381 is 8-fold the GDP per person of France. Such evidence constitutes an additional argument for health policy makers when making a case for increased investment to optimize IHR capacities, and coverage of essential health services, and safely managed water and sanitation services. The other rationales for increased investments in the development of resilient health-related systems include the fact that a pandemic, such as COVID-19, can trigger health systems and socioeconomic crises of significant magnitudes ^42^; and also the fact that every human being has the right to life ^35^.

## Data availability

All data underlying the results are available as part of the article and no additional source data are required.
