# Supplementary material for: The discounted value of human lives lost due to COVID-19 in France
Source: F1000Res. 2020 Oct 15;9:1247. [Version 1] doi: 10.12688/f1000research.26975.1 (PMC8082570; doi:10.12688/f1000research.26975.1)
Supplement: Peer review history

# Peer review: Reviewer response for version 1

**DOI:** 10.5256/f1000research.29797.r83336

**Published:** 2021-04-28

**Authors:** Neda Soleimanvandiazar

**Affiliations:** 1 Preventive Medicine and Public Health Research Center, Psychosocial Health Research Institute, Iran University of Medical Sciences, Tehran, Iran

**Introduction:**

- **Comment:** It would be better if the authors, in the introduction of the study explain the reason for the selection of 13 regions and 5 territories of France and as well how to select the desired regions (for example the features of these regions) that are given in the results section.

**Introduction: Paragraph 3**

- **Comment:** The reason for the explanation of the four factors that caused the relatively large number of deaths due to COVID-19 sustained by France is not specified in the third paragraph by the authors.

**Methods: Step 3**

 Discounting of the years of life lost (DYLL).

- **Comment:** The authors mentioned that the 3% discount rate was chosen because it has been used in our previous COVID-19 related economic studies, but it would be better to explain in a sentence about choosing a 3% discount rate with reference (e.g. In European countries, the discount rate is usually considered 3%, reference).

**Discussions**

 Contrasting of study findings with those from other countries:

- **Comment:** The discussion is not very well written. In the discussion, the authors merely cite the findings of the economic value per human life of their previous studies in this field and do not provide an explanation of the possible reasons for the differences in the economic value of human life between different countries. It is recommended that the discussion section of the article be upgraded.

Suggestions for further economic studies:

- **Comment:** Some suggestions are not in line with the results of this study.

Is the work clearly and accurately presented and does it cite the current literature?

Partly

If applicable, is the statistical analysis and its interpretation appropriate?

Yes

Are all the source data underlying the results available to ensure full reproducibility?

Yes

Is the study design appropriate and is the work technically sound?

Yes

Are the conclusions drawn adequately supported by the results?

Yes

Are sufficient details of methods and analysis provided to allow replication by others?

Yes

Reviewer Expertise:

Health service utilization, Inequality in Health services, Inequality in Health, Social Determinants of Health, Social support, Social Network, Social Health in the Elderly, AIDS & HIV, High risk Behaviors, Drug use and Substance use

I confirm that I have read this submission and believe that I have an appropriate level of expertise to confirm that it is of an acceptable scientific standard.

# Peer review: Reviewer response for version 1

**DOI:** 10.5256/f1000research.29797.r73179

**Published:** 2020-12-03

**Authors:** Patricia Akweongo

**Affiliations:** 1 Department of Health Policy, Planning and Management, School of Public Health, College of Health Sciences, University of Ghana, Legon, Ghana

**Specific Comments for the revision of the paper**

*Introduction Paragraph 4 line 1: *Second, the average of 13 International Health Regulations (IHR) core capacity score for France was 82 (on a scale of 0 to the target of 100) in 2019 ^7^^,^

**Comment**: The authors estimate the  average IHR core capacity score for France as 82 . It is unclear if this was an average of 13  items scored in Table 1 or how the authors estimated that. For summing up the 13 items in Table 1 under France the average is 74 instead of 82? This method for arriving at the 82 needs to be explained. 

*Introduction Paragraph 4 line 4-7:* The French points of entry capacity score of 40 were lower than the average score for the World Health 

**Comment:** This should read "The French points of entry capacity score of 40 was lower than the average score for the World Health" 

*Introduction Paragraph 4 line 4-7:*

**Comment**: The authors indicate that France point of entry  capacity score was 40 whereas that of EU was 61 representing 52.2%. It is unclear how the authors arrived at this percentage (52.2%)

*Introduction: Paragraph 3-4*

**Commen** **t:** In paragraph 3 the authors mentioned four factors  might explain the relatively large number of COVID-19 deaths sustained by France. The first being the more than 2 months  delay with restrictions (Paragraph 3), the second being the IHR capacity scores  (Paragraph 4) and then it repeats the second factor as in Table 2 as the general health system determinants. This is quite confusing as one loses track of the four factors that accounted for the state in which France found herself with respect to Cover-19. The authors will need to revise this to make it clear what these 4 factors are.

*Introduction Paragraph 3 line 2*

 First, there was more than two months’ delay in country-wide implementation of public health interventions that could have prevented (or slowed)

**Comment:** This should be revised to read  "First, there was more than two months’ delay in country-wide implementation of public health interventions that could have prevented or slowed " (since prevention is not the same as slowed)

*Methods*

 Step 3: Discounting of the years of life lost (DYLL).

**Comment:**The authors indicate here that the discount of 3% is chosen based on their previous work but they cite other articles as references as well. Thus, the article  should rather  indicate that "(a) A discount rate of 3% was chosen because it has been used in previous COVID-19 related economic studies ^13^^–^^21^, "

 Step 6: Calculation of the share of TDVHL accruing to the 13 regions and five territories of France ^29^ through multiplication of the TDVHL by proportion of COVID-19 deaths sustained by specific region and territory.

**Comment:**In the introduction of the study it was not indicated that 13 regions and 5 territories of France will be used to show the share of these to the TDVHL. It thus pops up here with a previous mention of that in the introduction or even in the methods where information on the study site could be described. The characteristics of these territories may affect the outcomes of the study.

 Step 7: A one-way sensitivity analysis was performed to evaluate the effect of changes in discount rate and the average life expectancy on the estimated TDVHL.

**Comment:** The authors chose to use the world average life expectancy and the world highest average life expectancy of 88.13 for women from Hong Kong for the sensitivity. Given that the authors began comparing France with the rest of EU, I was  expecting that the authors will first conduct the sensitivity analysis  using  average life expectancy for the Europe before comparing with the rest of the world.  

*Discussions*

*Contrasting of study findings with those from other countries:*

**Comment** **:**The authors refer only to their previous studies in comparing and contrasting their findings but I think the paper will benefit greatly by comparing and contrasting the findings with other studies in this same field as well.

*Recap of findings* 

 Reanalysis of the HCA model with the world average life expectancy dwindled the TDVHL by Int$7,750,187,267 

**Comment:**This should rather read" Reanalysis of the HCA model with the world average life expectancy reduced the TDVHL by Int$7,750,187,267 

*Limitations of Study*

 For instance, traditional HCA values YLL among children below 14 years ^38^, retired (62 years and above) ^38^, homemakers (not employed outside the home), unemployed, and severely handicapped.

**Comment**: This sentence above is incomplete with the use of "For instance....". Authors should consider revising this.

 Second, the current study did not compare the costs and benefits of a raft of alternative preventive community-level interventions implemented by the French Government and citizens to limit transmission of COVID-19.

**Comment:** This sentence above should read "Second, the current study did not compare the costs and benefits of a raft of alternative community-level interventions implemented by the French Government and citizens to limit transmission of COVID-19.

**General Comment:**This paper highlights issues that might explain the years of lives lost due to Covid-19 in France that may appeal to policy makers to make great investments in interventions needed to reduce deaths lost and increase GDP. More elaborately in this paper is the clear and robust description of the analytical methods for calculating the lives lost. This step-by-step methodological approach could easily be replicated in many countries and sites to produce more evidence that will guide policy makers in the prioritizing investments in Covid-19 to curb the pandemic.

Is the work clearly and accurately presented and does it cite the current literature?

Yes

If applicable, is the statistical analysis and its interpretation appropriate?

Yes

Are all the source data underlying the results available to ensure full reproducibility?

Yes

Is the study design appropriate and is the work technically sound?

Yes

Are the conclusions drawn adequately supported by the results?

Yes

Are sufficient details of methods and analysis provided to allow replication by others?

Yes

Reviewer Expertise:

Health Equity, Economic Evaluation, Health Systems, Health financing, Human resource for health, malaria, gender and violence , etc

I confirm that I have read this submission and believe that I have an appropriate level of expertise to confirm that it is of an acceptable scientific standard.
